# 

**Published:** 1910-06-11

**Authors:** 


### APPOINTMENTS.

The Droylsden District Council has appointed Dr. F. G. Pell-Ilderton medical officer of health. He was house surgeon at the Manchester Royal Infirmary.

Mr. Robert Donaldson, M.A., M.B.Ch.B., F.R.C.S. (Edinburgh), has been appointed by the Council of Sheffield University to the post of junior demonstrator in pathology, in succession to Dr. A. E. Bennes, deceased.

### GRADUATES' MEDICAL SCHOOLS.

DIARY FOR THE WEEK, JUNE 13 to JUNE 18.

**MEDICAL GRADUATES' COLLEGE AND POLYCLINIC,** 22 Chenies Street, W.C.

At 4 p.m.—**June 13**, Dr. H. G. Adamson, **Clinique (skin).**
5.15 p.m.—**June 13**, Sir John Broadbent, Bart., **Malignant Endocarditis.**
4 p.m.—**June 14**, Dr. Wm. Ewart, **Clinique (medical).**
5.15 p.m.—**June 14**, Mr. A. Carless, **Malignant Disease of Bone (lantern illustrations).**
4 p.m.—**June 15**, Mr. P. J. Freyer, **Clinique (surgical).**
5.15 p.m.—**June 15**, Mr. W. Stuart-Low, **Malignant Disease of the Nasal Passages: Its Pathology, Diagnosis, and Treatment.**
4 p.m.—**June 16**, Dr. C. Theodore Williams, **Clinique (medical).**
5.15 p.m.—**June 16**, Dr. Geo. Oliver (Harrogate), **Some Bedside Clinical Methods with Demonstrations and Examples.**
4 p.m.—**June 17**, Dr. St. Clair Thomson, **Clinique (ear, nose, and throat).**

**THE POST-GRADUATE COLLEGE,** West London Hospital, Hammersmith, W.

At 10 a.m.—**June 13** and **16, Demonstrations by Surgical Registrar.**
10 a.m.—**June 17, Demonstration by Medical Registrar.**
11.30 a.m.—**June 14, Demonstration of Minor Operations.**
12.15 p.m.—**June 15**, Dr. Grainger Stewart, **Practical Medicine.**
5 p.m.—**June 13**, Mr. Bishop Harman, **Acute Iritis.**
5 p.m.—**June 14**, Dr. Pritchard, **Clinical Pathology.**
5 p.m.—**June 15**, Dr. Morton, **X-ray Examination of the Urinary System.**
5 p.m.—**June 16**, Mr. Baldwin, **Practical Surgery. Lecture III.**
5 p.m.—**June 17**, Dr. Saunders, **Clinical Lecture with Cases.**

**LONDON SCHOOL OF CLINICAL MEDICINE,** Seamen's Hospital, Greenwich, S.E.

At 4 p.m.—**June 13**, Mr. Lake, **Operations for Acute Mastoid Disease.**
4 p.m.—**June 14**, Dr. James Taylor, **Some Clinical and Pathological Aspects of Nervous Disease.**

**THE HOSPITAL FOR SICK CHILDREN,** Great Ormond Street, W.C.

At 4 p.m.—**June 16**, Mr. Waugh, **Acute and Chronic Inflammatory Lesions of Bone.**

**ST. MARK'S HOSPITAL FOR DISEASES OF THE RECTUM,** City Road, E.C.

At 2.30 p.m.—**June 17**, Mr. F. C. Wallis, **Fissure and Pruritus.**

**LONDON HOMŒOPATHIC HOSPITAL,** Great Ormond Street, W.C.

At 5 p.m.—**June 17**, Dr. G. Burford, **The Medical Treatment of Gynæcological Cases.**

**NORTH-EAST LONDON POST-GRADUATE COLLEGE,** Prince of Wales's General Hospital, Tottenham, N.

At 4.30 p.m.—**June 14**, Mr. de Prenderville, **Anæsthetic Dangers and how to Avoid them.**

**THE BROMPTON HOSPITAL FOR CONSUMPTION AND DISEASES OF THE CHEST,** Fulham Road, S.W.

At 4 p.m.—**June 15**, Dr. Habershon, **Mitral Stenosis.**

**ROYAL SOCIETY OF MEDICINE,** 15 Cavendish Square, W. (Temporary address during building.)

At 5.30 p.m.—**June 14, Surgical Section:**
In the Large Theatre, Medical School, University College, W.C.
*Discussion* on "The present position of the treatment of syphilis." (Second Meeting.)
5 p.m.—**June 15**, Special Meeting of Fellows of the Society.
At Morley Hall, George Street, Hanover Square, W.
*Discussion* on "Vaccine Therapy; its administration, value and limitations" (fifth meeting). Re-opened by Mr. Deane Butcher, and continued by Dr. Lewis Jones, Dr. L. S. Dudgeon, Dr. David Lawson, Dr. Crace-Calvert, Mr. Leonard Noon, Dr. W. Parry Morgan, Dr. J. C. MacWatters.
5 p.m.—**June 16, Dermatological Section:**
At 11 Chandos Street, W., Clinical Cases.

**NATIONAL HOSPITAL FOR THE PARALYSED AND EPILEPTIC,** Queen Square, Bloomsbury, W.C.

At 3.30 p.m.—**June 14**, Sir Wm. Gowers, **Vertigo.**
3.30 p.m.—**June 17**, Dr. E. F. Buzzard, **Clinical Cases.**

**CENTRAL LONDON THROAT AND EAR HOSPITAL,** Gray's Inn Road, W.C.

At 3.45 p.m.—**June 14** and **17**, Dr. Andrew Wylie, **Larynx.**

---

Last week, Dr. Charles Walter Biden, of Laxfield, near Halesworth, was sued by a former patient for damages for negligence and unskilful treatment. Plaintiff's counsel stated that his client was formerly a solicitor's clerk, and when in September 1908 he returned to his home at Laxfield for a holiday he became ill. After recovering from pneumonia he was troubled with his knee, and Dr. Biden's *locum tenens* diagnosed the case as acute rheumatism. Dr. Biden on returning from his holiday also assumed that it was rheumatism, but on October 22 he declared that the plaintiff was suffering from blood poisoning. On October 31, as the knee was three times its normal size and as the plaintiff was suffering much pain, a neighbouring doctor—Dr. Ball—was called in, and he at once saw that pus had formed, and it was agreed that an operation was necessary. The swelling burst before this took place. Counsel said that before and after this happened proper treatment, as laid down in the most elementary text-books, had not been followed. Had the plaintiff received proper treatment, his leg when becoming stiff would not have remained so at an angle. Mr. C. D. Green, a surgeon, stated that he found the knee quite stiff, and the bone of the leg was displaced backward and outward. He contended that the proper treatment had not been followed for getting rid of the pus. Counsel for the defendant said that no question of want of care could arise, because no man could have bestowed more anxious care than Dr. Biden, who attended the plaintiff ninety-six times almost consecutively. Dr. Biden said that two days before it was decided to make an incision into the knee it burst, and an operation was therefore considered to be unnecessary. He had no reason to expect the existence of pus until October 28. Four other medical men who gave evidence in the case agreed with this opinion. The jury returned a verdict for the defendant, for whom judgment was entered with costs.

### Gifts and Bequests.

Mr. John Tempest, of Duffield, Derby, has bequeathed £250 to the Derbyshire Royal Infirmary.

Mr. Frederick Charles Purchase, of Swansea, chief cashier, has left £200 to the Swansea Hospital.

Mr. William Harding, of Darlington, sharebroker, has left £500 to the Darlington Queen's Nurses' Association.

The late Mr. Thomas Windsor, M.R.C.S., of Great Budworth, Cheshire, has bequeathed £100 to the Mauldeth Hospital.

The Mercers' Company has voted 1,000 guineas to St. Bartholomew's Hospital as a memorial to the late King Edward VII.

Leicester Infirmary has received from the executors of the late Mr. Edmund Shortland the legacy of £50 bequeathed by his will.

A fund has been opened in Birmingham to erect a statue of the late King, and to raise a memorial fund for the rebuilding of the Children's Hospital.

Colonel Cecil Lennox Peel, of Wokingham, late Scots Fusiliers, has bequeathed £100 each to the Sussex County Hospital, Brighton, and the Royal Berks Hospital, Reading.

Prince Francis of Teck acknowledges £500 from Lord Strathcona, £250 from Mr. J. Bland Sutton, F.R.C.S., and £100 from Mr. Graham Prentice in response to his appeal for funds for the Middlesex Hospital.

The Trustees of Smith's (Kensington Estate) Charity have sent £536 to the Hospital for Women, Soho Square, for the Rebuilding Fund. The building, it is hoped, will be ready for occupation by patients in July.

The Treasurer of the Leicester Infirmary has received from Mr. W. B. Stock, of the Leicester Co-operative Society, the sum of £100, being a contribution from a lady, who desires to remain anonymous, to the General Funds of the Institution, in memory of her son "A. E. S."

The letting of seats to view the Funeral Procession at Messrs. Boots', Edgware Road, realised £281 11s., of which (minus the Marylebone Borough Council claim of £25 4s. for the licence) the balance of £256 7s. has been sent to the London Hospital, People's Subscription Fund.

Among the recent contributions to King Edward's Hospital Fund for London "In Memoriam King Edward VII." are the following: Women of Cornwall "One and All," £72 4s. 10d.; the J.P. Restaurants, Limited (being the profits of nineteen branches opened on the day of King Edward's funeral), £45 11s. 6d.; Miss C. Mocatta, £20; the Misses A. and F. Thomas, £15; the inhabitants of Lyndhurst, Emery Down and Bank (New Forest), £14 6s.

The Treasurer of the Leicester Infirmary has received from Mr. George Sneath, of Hendon, a cheque for £100 as a donation to the Building Fund of that institution in memory of the late Miss Charlotte Perkins, of Leicester. It is gratifying to record that this sum was a legacy bequeathed to Mr. Sneath under the will of Miss Perkins, which he has generously devoted to the General Hospital for the Counties of Leicester and Rutland.

Mr. George Trenchard Cox, of Eaton Gardens, Hove, of the firm of general produce merchants, has bequeathed £100 each to St. Mary's Hospital, Paddington; the North-West London Hospital; King's College Hospital; the Dental Hospital, Leicester Square; the Royal Free Hospital, Gray's Inn Road, W.C.; the London Hospital; and the Metropolitan Hospital, Kingsland Road, N.E.; and £50 each to the Poplar Hospital and the Brompton Hospital for Consumption.

### Jottings.

The Queen has sent a gift of flowers to the Middlesex Hospital, St. Bartholomew's Hospital, the Friedenheim Hospital, Swiss Cottage, and to the Hospital for Women, Soho Square.

St. Vincent's Cripples' Home, Clarence Road, Clapham Park, S.W., will be opened on June 6, 1910, at 3.30 p.m., by the Lord Mayor, in the presence of the Most Rev. the Archbishop of Westminster. After the opening ceremony the Home will be open for inspection.

Lord Kenyon, in presiding at a meeting of the executive committee of the County Association for the Prevention of Consumption at Shrewsbury, announced that King George had consented that the proposed Shropshire Consumption Sanatorium should be erected as a memorial to the late King Edward.

The fifth annual general meeting of the Convalescent Homes Association, 32 Sackville Street, Piccadilly, W., will be held in the Committee Room on the 9th inst., at 4.30 p.m., Sir William S. Church, Bart., K.C.B., in the chair. The report for the year 1909 will be submitted, and the Honorary Secretary will explain the object and work of the Association.

The Meath Home of Comfort for Epileptics, Godalming, Surrey, will on Saturday, June 11, 1910, hold the anniversary festival of the opening of the Home. The festival service will be held in Godalming Parish Church at 3 p.m. Preacher, the Right Rev. the Lord Bishop of Lewes. Offertory for the funds of the Meath Home. Patients' industries for exhibition and sale at 4 p.m. Tea and coffee stall.

The Mercer's Hospital, Dublin, which, having been founded in 1734, is the oldest hospital in Ireland, has issued an appeal to the Irish men and women in this country to help it in its present financial straits. It is proposed to found cots in memory of King Edward. We feel sure that if the late King's visits to Ireland and his interest in Irish hospitals were made public by the committee this appeal would be responded to quickly enough.

The annual distribution of prizes and certificates gained by the nursing staff of the Leicester Infirmary was held in the Nurses' Recreation Room of the new Home on Tuesday last. Sir Edward Wood presided over a large attendance, which included the Mayor and Mayoress of Leicester, Lady Hazlerigg, Dr. F. M. Pope, Mr. C. J. Bond, Mr. G. C. Franklin Dr. Seveshe, and Mr. Cecil Marriott (Examiners), Mr. T. Fielding Johnson, jun., and Miss G. A. Rogers (Lady Superintendent). The prizes and certificates were presented by Lady Hazlerigg (wife of the Vice-Chairman of the Board, Sir Arthur Hazlerigg).

Mr. Sydney Holland has received the following letter in reply to a vote of condolence passed by the House Committee and medical staff of the London Hospital: "Buckingham Palace, May 11, 1910.—Dear Mr. Holland, Queen Alexandra has received your letter on behalf of the committee of the London Hospital and also of the Medical Council. Her Majesty is most deeply touched by their expressions of sympathy, and desires me to ask you to kindly convey her heartfelt thanks to one and all who have associated themselves in this kind thought of her in her terrible bereavement.—Believe me, yours sincerely, Sydney Greville.

### TO HOSPITAL SECRETARIES AND OTHERS.

We invite contributions from any of our readers, and shall be glad to pay, according to length, for items of news for the institutional section (including appointments, resignations, gifts, etc.) which we may publish. All payments for manuscripts used are made as early as possible after the beginning of each quarter.

### COMING EVENTS OF THE WEEK.

Saturday, June 11.—Anniversary Festival, Meath Home for Epileptics, Godalming, 3 p.m.
University of Leeds, Chancellor's Installation.
Belgrave Hospital for Children, Clapham Road, Pound Day, opening by Lord Plymouth, 2.45.
Commemoration Day, Livingstone College, Leyton, E., 3 p.m.

Sunday, June 12.—HOSPITAL SUNDAY.
Collections in all the Churches in aid of Hospitals.
Visiting-day at Hospitals.

Tuesday, June 14.—Dr. Andrews' Croonian Lecture, Royal College of Physicians, Pall Mall, S.W., 5 p.m.
League of Mercy, N.-E. Sussex District, Garden Meeting at Old Lodge, Ashdown Forest, 3 p.m.

Wednesday, June 15.—Harben Lecture, Institute of Public Health, Sir Wm. Leishman, 7 p.m.
Annual Meeting, Factory Girls' Holiday Fund, Aubrey House, Campden Hill, 5 p.m.
League of Mercy, N.-E. Sussex District, Mrs. Bruce, "At Home," Burraland, Heathfield, 3.30 p.m.

Thursday, June 16.—Dr. Andrews' second Croonian Lecture, Royal College of Physicians, Pall Mall, S.W., 5 p.m.

Friday, June 17.—Tropical Medicine, Society of, Annual Meeting.

### The Research Defence Society.

The Research Defence Society, which was founded two years ago "to make generally known the facts as to experiments on animals in this country," held its annual meeting on Monday last in the library of the Royal College of Physicians, Pall Mall East, Lord Cromer presiding. Mr. Sydney Holland presented the annual report. This showed that the members and associates had increased by 800 to 3,360. Commenting on the report, Mr. Holland remarked that the opening of anti-vivisection shops had proved the best advertisement for the Society, which took the opportunity to circulate its own literature in the same neighbourhoods. No Englishman could fail to be disgusted with the recent circulation of a pamphlet ascribing the King's death to knowledge gained by experiments on animals—even if that had been true. The Society wished to defend not cruelty, but truth—to show what good had been done by experiments on animals and in what a merciful way they were carried on here. Sir Douglas Powell said it was deplorable that the Society should have to protect the public from the machinations of the clique now dominating and misguiding another association, whose proper work would be to supplement the efforts of science, to diminish suffering, and prolong life. The Society for the Prevention of Cruelty to Animals had done much to ameliorate the conditions of animals, but had unfortunately entered on a wild crusade against science. Sir David Bruce referred to the victories gained by animal experiments over tropical diseases which had destroyed vast numbers of human lives. Lord Cromer said that in this matter he had to follow the example of Cobden, who said: "The only way to get an idea into the head of the British public is to go on repeating the same thing over and over in different language." The men who carried on research by experiment could show a noble record, which the public should be asked to compare with the sterility of their opponents. He regretted the delay in issuing the report of the Royal Commission, which would probably be published next month.

### AN ACTION FOR DAMAGES AGAINST A DOCTOR.

In the London Sheriff's Court last week the case of Pratt *v.* Fletcher, remitted from the High Court for the assessment of damages, was heard. The case arose out of the death of Mrs. Pratt, after taking a bottle of medicine supplied by the defendant, Dr. John Fletcher. At the inquest the jury returned a verdict of "Death by misadventure." The action was brought by Mr. Pratt to recover damages for the death, through the defendant's negligence, of Mrs. Pratt. The details of the circumstances attending the death of Mrs. Pratt have already been reported in The Hospital. Dr. M. J. Williams, who gave evidence on behalf of the plaintiff, said that on December 31 he was called to see Mrs. Pratt, and found her dead. He tested the contents of Dr. Fletcher's medicine bottle, and found that it contained strychnine. Subsequently he saw Dr. Fletcher, who said, "There is nothing in it; the woman had been ill for some time. She died of a weak heart." The witness rejoined, "But the body is rigid," and Dr. Fletcher replied, "Oh; that's rigor mortis." The witness pointed out that rigor mortis did not set in so soon as that in a natural death. Dr. Fletcher said the patient had been suffering from diarrhœa. The witness asked him whether he suggested ptomaine poisoning, and he answered, "Yes, ptomaine poisoning; that's right." The witness said that the symptoms pointed to strychnine poisoning. Dr. Fletcher said, "I gave her some strychnine for her weak heart." The witness told him that the case would have to go before the Coroner, and Dr. Fletcher said he was prepared to give a death certificate. While the witness was showing Dr. Fletcher out he heard the latter tell Inspector Elliott that death was due to a weak heart, consequent upon influenza and diarrhœa and ptomaine poisoning. Inspector Elliott confirmed Dr. Williams's evidence, and said if Dr. Williams had not been called in it was quite possible that Mrs. Pratt might have been buried on Dr. Fletcher's certificate. The defendant said he had admitted that the woman died from strychnine poisoning through his inadvertence. He wished to express his profound regret at what had happened. When he told Inspector Elliott that the woman died from ptomaine poisoning, that was his opinion at the time. The jury found for the plaintiff, and assessed the damages at £200.

### The Institution Library.

"Burdett's Hospitals and Charities," 1910. (Net, post free.) ... ... ... ... ... ... ...10s. 11d.
"Hospital Expenditure: The Commissariat." For the use of Managers. ... ... ... ... ... 2s. 6d.
"The District Nursing Association; Hints on how to Start it." (Net, post free.) ... ... ... 1s. 1d.
"A Legal Handbook for the use of Hospital Authorities." .. ... ... ... ... ... ... 2s. 6d.
Ledgers, Charts, Case-papers, and every kind of Institutional Literature.

Of all booksellers or of The Scientific Press, Limited, 28 and 29 Southampton Street, Strand, London, W.C.

THE HOSPITAL. June 11, 1910.
*Name* ..............................
*Address* ..............................
..............................
This Coupon must accompany manuscript or contributions intended for The Hospital.

